# Antepartum fetal heart rate feature extraction and classification using empirical mode decomposition and support vector machine

**DOI:** 10.1186/1475-925X-10-6

**Published:** 2011-01-19

**Authors:** Niranjana Krupa, Mohd Ali MA, Edmond Zahedi, Shuhaila Ahmed, Fauziah M Hassan

**Affiliations:** 1 Department of Electrical Electronic and Systems Engineering, Universiti Kebangsaan Malaysia, Bangi, Malaysia; 2 School of Electrical Engineering, Sharif University of Technology, Tehran, Iran; 3 Department of Obstetrics and Gynecology, Universiti Kebangsaan Malaysia Medical Center, Cheras, Malaysia; 4 Department of Obstetrics and Gynecology, Cyberjaya University College of Medical Sciences, Cyberjaya, Malaysia

## Abstract

**Background:**

Cardiotocography (CTG) is the most widely used tool for fetal surveillance. The visual analysis of fetal heart rate (FHR) traces largely depends on the expertise and experience of the clinician involved. Several approaches have been proposed for the effective interpretation of FHR. In this paper, a new approach for FHR feature extraction based on empirical mode decomposition (EMD) is proposed, which was used along with support vector machine (SVM) for the classification of FHR recordings as 'normal' or 'at risk'.

**Methods:**

The FHR were recorded from 15 subjects at a sampling rate of 4 Hz and a dataset consisting of 90 randomly selected records of 20 minutes duration was formed from these. All records were labelled as 'normal' or 'at risk' by two experienced obstetricians. A training set was formed by 60 records, the remaining 30 left as the testing set. The standard deviations of the EMD components are input as features to a support vector machine (SVM) to classify FHR samples.

**Results:**

For the training set, a five-fold cross validation test resulted in an accuracy of 86% whereas the overall geometric mean of sensitivity and specificity was 94.8%. The Kappa value for the training set was .923. Application of the proposed method to the testing set (30 records) resulted in a geometric mean of 81.5%. The Kappa value for the testing set was .684.

**Conclusions:**

Based on the overall performance of the system it can be stated that the proposed methodology is a promising new approach for the feature extraction and classification of FHR signals.

## Background

**Figure 1:**
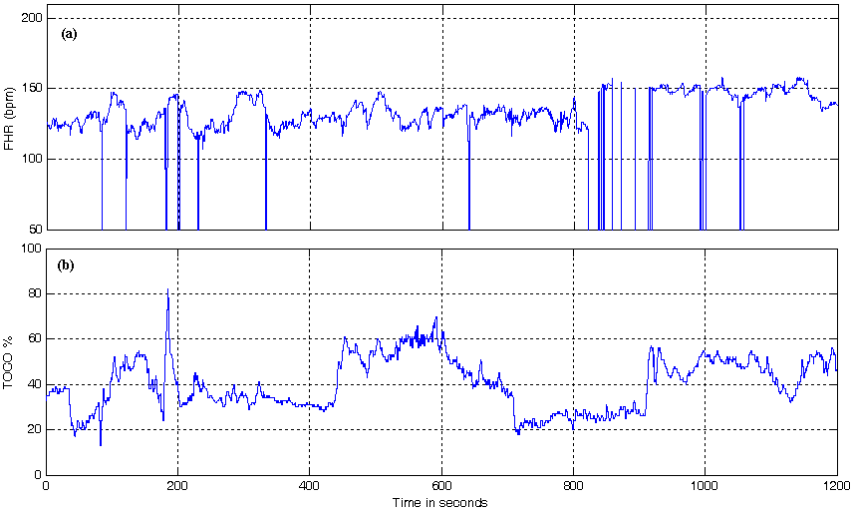
**Cardiotocogram**. Typical CTG: (a) The FHR signal and (b) The UA signal

Cardiotocograph (CTG) is a graphical representation of fetal heart rate (FHR) and uterine activity (UA), also termed as electronic fetal monitoring, and has been an indispensable part of antepartum and intrapartum fetal surveillance [1] for four decades. A typical CTG is depicted in Figure 1. Heart rate (HR) contains reliable information about the synergic activity of the autonomic nervous system (ANS) that regulates the heart beat dynamics [2]. Parameters from the HR signal provide interesting hints about the generation of disease conditions and hence can be used to differentiate pathological states [3]. The analysis of FHR signal used in monitoring the fetal well being is a powerful tool in establishing the development of the nervous system of the fetus during the last period of pregnancy, starting from the 25^th ^week of gestation [4]. However, the current clinical practice of visual interpretation of CTG shows a high degree of inter-observer and intra-observer variability [5] due to its large dependency on the expertise and experience of the clinician(s) involved [6]. Advances in signal processing and pattern recognition techniques and skepticism over inconsistency in FHR interpretation paved the way for computerized methods [7]. The computerization of non-stress-test (NST) involves two principal problems namely, feature extraction and classification, and subsequent interpretation [6]. It is important to mention that the interpretation phase requires a contextual analysis of all the physiological, pathological and clinical aspects needed in assessing the well being of the fetus.

Diverse approaches have been investigated using conventional and artificial intelligence techniques for feature extraction [3,8-13] and also to come out with diagnostic systems. Computerized CTG analysis systems were developed [14-16] adhering to the guidelines issued by the International Federation of Obstetrics and Gynecology (FIGO) [17]. A non-linear back propagation artificial neural network (ANN) was introduced for the interpretation of NST records [18] and around the same time a software program to predict fetal acidosis at birth was also developed [19]. ANN was used to distinguish between the normal and pathological fetal conditions [20,21]. An expert system [22] capable of handling uncertainties in FHR interpretation was introduced, which was later transformed to a fuzzy system [23]. Yet again, an expert system called NST-EXPERT [24,25] was developed and was later named computer aided fetal evaluator (CAFÉ) system [6]. A method using wavelet analysis and self organizing map was developed to diagnose fetal hypoxia based on the scale-dependent features extracted from the FHR [26]. Another method based on wavelet transform and cumulative holder exponent was proposed [27] for real-time fetal monitoring during labor. In [1] scale-dependent features extracted from wavelet components of the FHR signal were categorized using a support vector machine (SVM). SVM classifier was employed yet again to predict the risk of metabolic acidosis in newborns [28], to detect fetal distress [29,30] and to discriminate healthy fetuses from the ones with hypoxia [31]. Recently, a new approach based on FHR variability analysis involving Lempel Ziv complexity index and multiscale entropy was proposed for the early identification of intrauterine growth-restricted (IUGR) fetuses [32].

The above discussion shows that although some approaches have shown promising results, none has been widely accepted and there is still room for improvement [1,28] to reach the ultimate goal of a completely reliable method to assess fetal well being with minimal intervention from obstetricians. In an effort to achieve this goal we propose an innovative approach in this paper for FHR classification combining empirical mode decomposition (EMD) and SVM techniques.

EMD has been used in several biomedical applications (introduced for the study of ocean waves in 1998 [33]) such as artifact reduction in electrogastrogram and to extract the lower esophageal sphincter pressure in the gastro-esophageal reflux disease [34,35]. There has been a noticeable contribution from EMD based methods in processing electrocardiogram (ECG) signals [36,37]. It has also been used in the analysis of heart rate variability (HRV) [38] and high frequency FHR variability [39]. In addition to the above applications EMD has been employed for CTG signal enhancement [40].

SVM is a powerful supervised machine learning tool introduced recently in the framework of statistical learning theory [41]. It is used in a number of applications for both pattern classification and non-linear regression [42-44]. It has the ability to generalize well on unknown data without requiring the domain knowledge, and even when the sample size is small [45] which make it an attractive solution in difficult pattern recognition problems. Here, statistical features are extracted from FHR signals using EMD and later classified using the SVM classifier.

## Methods

**Figure 2:**
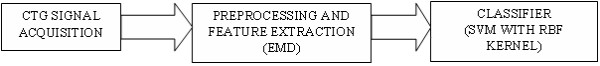
**Proposed methodology**. Stages involved in the proposed methodology

A brief explanation on the two important techniques (EMD and SVM) employed in this work is provided in the beginning of this section. Later a detailed description of the proposed methodology for the extraction of statistical features and classification of FHR signals as 'normal' and 'at risk' (Figure 2) is given. Finally, the four important stages in this work, namely, data acquisition, preprocessing, feature extraction and classification, are defined.

### Empirical Mode Decomposition

EMD, proposed by Huang *et al *[33], is a method to decompose non-linear and non-stationary time series into several monotonic components termed as intrinsic mode functions (IMF) of different time scales. The most appealing nature of EMD is its dependency on the data-driven mechanism which does not require a priori known basis unlike Wavelet and Fourier transform.

The EMD method identifies all the local maxima and minima for a given input signal *x(t) *which are connected by spline curves to form the upper and the lower envelopes, *e*_up_*(t) *and *e*_low_*(t)*, respectively. The mean of the two envelopes is calculated as *m(t) *= [*e*_up_*(t) *+ *e*_low_*(t)*]/2 and is subtracted from the signal using q*(t) *= *x(t) *- *m(t)*.

An IMF *c*_*i*_*(t) *is obtained if q*(t) *satisfies the two conditions of IMF, these are, the number of extrema and number of zero crossings is either equal or differs at most by one, and the envelopes defined by the local maxima and minima are symmetric with respect to zero mean. This procedure is called as the sifting process. Then *x(t) *is replaced with the residual *r(t) = x(t)-q(t)*. If *q(t) *is not an IMF, *x(t) *is replaced with *q(t)*. The above process is repeated until the residual satisfies the stopping criterion called sum of difference, S_D _shown in equation (1), which is generally set between 0.2 and 0.3.

(1) $$S_{D}=\sum_{t=0}^{T}\frac{|q_{k-1}(t)-q_{k}(t){|}^{2}}{q_{k-1}^{2}(t)}$$

At the end of this process the signal *x(t) *would result in *N *IMFs and a residue signal as in equation (2).

(2) $$x(t)=\sum_{n=1}^{N}c_{n}(t)+r_{N}(t)$$

Where *n *represents the order of IMFs, *n = 1 *to *N *and *r*_*N *_denotes the final residue which can also be considered as an IMF, shown in equation (3).

(3) $$x_{d}(t)=\sum_{i=1}^{N_{f}}c_{i}(t)$$

Here, *N*_*f *_is the total number of IMFs including the residue. The signal *x(t) *is decomposed such that the lower-order components represent fast oscillation modes and higher-order components represent slow oscillation modes. A detailed explanation of the method is provided in [40].

### Support Vector Machine

SVM is a supervised learning tool that can be used for pattern classification. The main goal of SVM is to construct an optimal hyperplane as the decision surface in such a way that the margin of separation between the closest data points belonging to different classes is maximized. SVM is based on the principle of structural risk minimization method [46]. In a binary classification problem which is of interest in this work, each one of the set of points belongs to either one of the two classes.

Consider a training set ${\left\{(x_{i},d_{i})\right\}}_{i=1}^{l}$ where *x*_*i *_is the input pattern for the *i*^*th *^sample and *d*_*i *_∈{-1,+1} is the corresponding desired output and *l *is the number of observations. If the input patterns belonging to two different classes are linearly separable then there exists a hyperplane that maximizes the margin of separation. For the optimal hyperplane the Euclidian norm of the weight vector *w *is minimum and at the same time satisfies the constraints in equation (4).

(4) $$d_{i}(w^{T}x_{i}+b)\geq 1\;\;\;\forall_{i}$$

This is a constrained quadratic optimization problem that may be solved using the method of Lagrange multipliers. Pattern classification problems in real life are not linearly separable. Here, SVMs depend on two mathematical operations: non-linear mapping of an input vector into a high-dimensional feature space and construction of an optimal hyperplane for separating the features.

Non-linear mapping is performed in accordance with the Cover's theorem [46] on the separability of patterns. Non-linearly separable patterns in the input space when transformed to a high dimensional feature space, they can be linearly separable with high probability. Therefore for each input pattern vector x_i _in the m_0 _dimensional input space we define a vector consisting of a set of real-valued functions{*ψ*_*i*_(*x*) | *i *= 1,2,..*m*_1_, as shown by *ψ*(*x*) = [*ψ*_1_(*x*),*ψ*_2_(*x*),.......*ψ*_m__1_]^T ^that map the m_0 _dimensional input points to m_1 _dimensional new feature space. An optimal hyperplane in the feature space is found as in equation (5).

(5)  Minimizing 12wTw+C∑i=1nζisubject to: di(wTψ(xi)+b)≥1-ζi and ζi≥0  ∀i

*ζ*_*i *_are called slack variables, a set of non-negative scalar variables; they measure the deviation of a data point from the ideal condition of pattern separability. Parameter *C *is a user specified positive value that controls the trade-off between maximizing the margin and minimizing the error. *ψ*(*x*_*i*_) is the non-linear mapping of input patterns from input space to feature space. The optimal discriminating function is given by equation (6).

(6) $$f(x)=sign\left[\sum_{i=1}^{n}d_{i}\alpha_{i}(\psi^{T}(x_{i})\psi (x))+b\right]$$

The coefficients *α*_*i *_are derived from the maximization of dual Lagrangian as in equation (7).

(7) $$\begin{array}{l} Q(\alpha )=\sum_{i=1}^{n}\alpha_{i}-\frac{1}{2}\sum_{i=1}^{n}\sum_{j=1}^{n}\alpha_{i}\alpha_{j}d_{i}d_{j}(\psi^{T}(x_{i})\psi (x_{j}))\\ \text{ subject to: }\sum_{i=1}^{n}\alpha_{i}d_{i}=0\\ \text{ and}\;\;0\leq \alpha_{i}\geq C\;\;\forall_{i} \end{array}$$

The points for which *α*_*i *_> 0, are called support vectors. The term *ψ*^*T *^(*x*_*i*_)*ψ*(*x*_*j*_) represents the inner product of two vectors in the feature space. We may introduce the inner-product kernel denoted by*K *(*x*_*i*_,*x*_*j*_), written as shown in equation (8).

(8) $$K(x_{i},x_{j})=\psi^{T}(x_{i})\psi (x_{j})$$

A kernel function is a function in the input space and hence, we may use the inner-product kernel *K *(*x*_*i*_,*x*_*j*_)to construct the optimal hyperplane without having to perform explicitly the non-linear mapping [42,46].

For classification problems dealing with medical data where the numbers of data in different classes are unbalanced, some researchers [1,28,47] have proposed the use of different penalty parameters in the SVM formulation as in equation (9).

Minimizing,

(9) $$\begin{array}{l} \frac{1}{2}w^{T}w+C^{-}\sum_{i;y_{i}=-1}^{n}\zeta_{i}+C^{+}\sum_{i;y_{i}=+1}^{n}\zeta_{i}\\ \text{ subject to:}\;d_{i}(w^{T}\psi (x_{i})+b)\geq 1-\zeta_{i}\\ \text{ and }\zeta_{i}\geq 0\;\;\forall_{i} \end{array}$$

C^+ ^and C^- ^are the penalty parameters used to penalize more heavily the undesired type of error, and the errors related to the class with the smallest population [47].

### Data Acquisition

**Figure 3:**
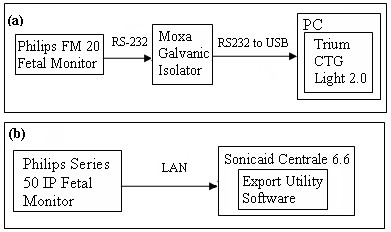
**Data acquisition setup**. Setups for acquiring CTG signals with (a) normal patterns and (b) abnormal patterns

CTG is a routine non-invasive fetal monitoring tool based on ultrasound Doppler combined with an external pressure transducer to record uterine activity. In this technique a transducer placed on the mother's abdomen transmits an ultrasound beam towards the fetal heart. The FHR is derived from the Doppler shifted echoes created by contractions of the fetal heart. An autocorrelation method is used to compare successive heart signals and test for similarity. CTG signals used in this research work were recorded at Universiti Kebangsaan Malaysia Medical Center (UKMMC). Data acquisition was carried out with the approval of UKMMC's ethical committee and after obtaining informed consent from all subjects. All 15 subjects were with singleton pregnancy and gestation age ranging from 34 weeks to 40 weeks. Since, the proposed study required both normal and abnormal patterns of FHR we had two different setups. The CTG signals with normal patterns were recorded from the day care clinic using an antepartum fetal monitor (Philips FM 20) and a software (Trium CTG Light 2.0 from Trium Analysis Online GmbH) as in Figure 3a. A galvanic isolator was used between the fetal monitor and the computer for safety purpose. CTG signals with abnormal patterns were obtained from the archived data on a Huntleigh server (Sonicaid™ Centrale, Labour Management System) at UKMMC, using the Export Utility Software as shown in Figure 3b. These data were recorded with the help of a fetal monitor (Philips Series 50IP). All the signals acquired using the above mentioned commercially available softwares had a sampling frequency of 4 Hz.

### Pre-processing and Feature Extraction using EMD

#### Preprocessing

**Figure 4:**
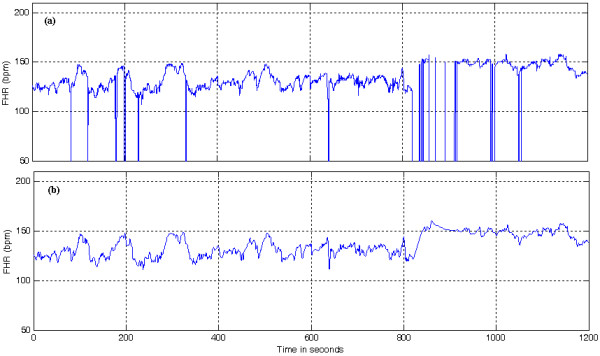
**Illustration of FHR signal enhancement**. (a) FHR signal with artifacts and (b) FHR signal after the removal of artifacts

Recorded FHR signals may possess missing beats and spiky artifacts (Figure 4a) due to the displacement of the transducer because of maternal or fetal movement and stress induced after the onset of labour. These artifacts are generally present and difficult to eliminate from the source. Missing beats in FHR can be about 20% - 40% of the data, especially during the final stages of labour [28,40]. For this reason, the quality of the FHR signal is estimated based on the number of missing beats and a poor quality signal is not subjected to further analysis [40]. In this work, missing beats are removed using a recursive algorithm and high frequency noises are suppressed using a method based on EMD explained in [40]. Once missing data segments are eliminated, the FHR signal is decomposed using EMD into several monotonic components (IMFs) as explained earlier. It is reported that the lower order IMFs which may contain noise have zero mean [36,40,35]. Hence, a statistical t-test is used to determine the high frequency noise components which are then separated from the signal in the EMD domain (equation (10)).

(10) $$\begin{array}{l} H_{0}:mean(c_{P_{M}S}(t))=0\\ H_{1}:mean(c_{P_{M}S}(t))\neq 0 \end{array}$$

Where, $c_{P_{M}S}(t)$ is the *M*th-order partial sum of the IMFs.

This test is applied first on the lowest order IMF and later on the partial sum $c_{P_{M}S}(t)$for *M *= 1, 2,... as shown in equation (11), until a partial sum $c_{P_{t}S}(t)$ is obtained, where the mean significantly deviates from zero.

(11) $$c_{P_{M}S}(t)=\sum_{i=1}^{M}c_{i}(t)$$

The above procedure indicates the number of IMFs that can be considered as noise, *P*_*t *_(noise order). Since this technique might result in over smoothing of the FHR signal, the noise order is estimated using equation (12) [40].

(12) $$P_{f}=\min(P_{t},3)$$

Then, the first *P*_*f *_components are eliminated from the set of IMFs for a given FHR signal. A denoised FHR signal (Figure 4b) can be obtained by applying the partial construction method on the remaining components.

#### Feature Extraction

**Figure 5:**
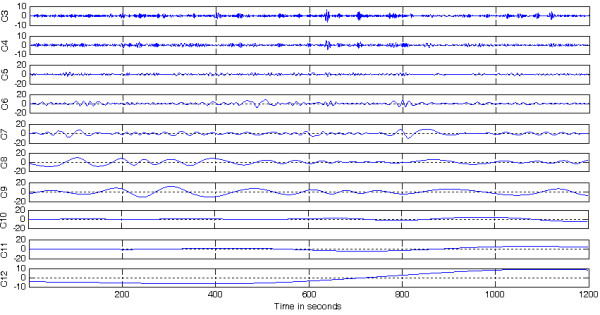
**Decomposed components after signal enhancement**. Remaining components (*C3-C12*) of a decomposed FHR signal used for statistical feature extraction, after the removal of noisy lower order IMFs (*C1 and C2*) based on *P*_*f*_.

For each of the remaining components (Figure 5) a standard deviation is estimated (equation (13)).

(13) SD(Ci)=[1N-1∑j=1N[Ci,j-M(Ci)]2]12for i=Pf+1 to N

These standard deviations (noise order, *P*_*f*_+1 onwards) are considered as statistical features of the FHR signal and are used as inputs to the SVM classifier. A similar approach has been employed in extracting statistical features from the wavelet coefficients, obtained from discrete wavelet transform (DWT) [1,48,49].

### Classification using SVM

#### SVM Classifier

The classification stage follows the feature extraction stage. The main objective of this stage is to classify the FHR signals as normal or at risk represented as '+1' and '-1' respectively. The entire process involves a training set and a testing set of data instances. A training set consists of features extracted from the decomposed FHR signal, also called the attributes, and the labels '+1' (normal) or '-1' (at risk) which are the target outputs. SVM should produce a model to classify the data instances in the testing set which consists of only features. The classification depends on the inner-product kernel used that produces different learning machines and hence different decision boundaries [46]. In this work, based on the statistical features extracted from FHR signals the radial basis function (RBF) kernel was used (equation (14)) to obtain reliable results. Parameters *C *and *γ *are specified by the user.

(14) $$k(x_{i},x_{j})=\gamma \exp{\left(\left|x_{i}-x_{j}\right|\right)}^{2}$$

The following procedure was employed during the classification stage:

(a)	Scaling the training and testing data sets.

(b)	General grid search method is considered an intractable problem and estimating accuracy for all possible combinations of *C *and *γ *is a time consuming process. Therefore, exponentially increasing values were considered initially to find a better possible set of values for *C *and *γ *that yielded better accuracy. Finally, *C *and *γ *values thus obtained are varied slightly to gain the best possible accuracy. The estimated set of values for *C *and *γ *are 4 and 2, respectively.

(c)	Training SVM using the chosen *C *and *γ *values to achieve the best cross-validation accuracy (CSV) possible.

(d)	Predicting the output of the testing set.

(e)	Estimating the accuracy of classification.

Since unbalanced data are used in this work the ratio of C^+^/C^- ^are set to the inverse of the corresponding cardinalities of the classes.

#### Description of the Data Set

A total of 129 FHR signals of 20 minutes duration were collected from pregnant women at gestation ages of 34 to 40 weeks. Missing beats were eliminated from all signals using the recursive algorithm. CTG traces were submitted to two experienced obstetricians (20 years in practice) for visual inspection who were asked to classify the traces as 'normal' (+1) or 'at risk' (-1). From the 129 records 29 showed disagreement between experts, therefore were eliminated. Out of the remaining 100 signals, 90 (30 normal and 60 at risk) signals were considered for the purpose of training and testing so that the normal and at risk signals ratio can be maintained at 1:2 during training and also in estimating the cross-validation accuracy. Hence, the proposed methodology was tested on 90 FHR recordings having mutually agreed interpretation from two experienced obstetricians.

From the 90 FHR signals in the data set, *Dataset*, a training set and a testing set consisting of 60 and 30 data instances, respectively, were created. The training set had 20 'normal' and 40 'at risk' data instances and the testing set was composed of 10 'normal' and 20 'at risk' samples. The training set was further divided into five subsets, as explained later in the results section, in order to obtain more reliable results. Data instances in the *Dataset *consisted of a maximum of 10 statistical features (standard deviation) extracted from the remaining decomposed components (IMFs) of the FHR signal, after the separation of noisy components (preprocessing method). Since the number of remaining decomposed components of 90 FHR signals in the set varied from 7 to 10, a maximum of 10 input features or parameters were considered. Features in the *Dataset *extracted from 90 FHR signals were used as inputs to the SVM classifier.

## Results

**Figure 6:**
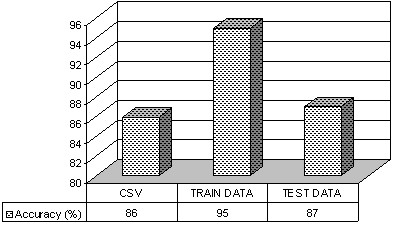
**Estimated accuracy**. Representation of accuracy obtained from the 5-fold cross validation (CSV), training set and the testing set for the *Dataset*

As a first step of validation a 5-fold cross validation method was employed using the FHR signals in the training set. This method is used for validating the systems, especially, when there is small number of data [50,1,49]. In this process, the training set was further divided into 5 non-overlapping subsets of 12 signals each. All the 5 sets had 4 instances that belonged to the 'normal' group and 8 from the 'at risk' group. The SVM classifier was trained with 4 subsets out of 5 and the 5^th ^subset was used as the validation set and the procedure is repeated 5 times in order to find the 5-fold cross validation accuracy. The values for (C, γ) parameters are found using a systematic grid search method [1]. Because of the unbalanced data set used in this work the penalty parameters (C^+^/C^-^) ratio was maintained to be the inverse of the corresponding cardinalities of the classes (1/20/40). A 5-fold cross validation accuracy of 86% was obtained for the *Dataset *as shown in Figure 6.

In the next stage of evaluating the system performance, we used both the testing set and the training set and estimated the accuracy of prediction. The testing data set prediction accuracy was 87% and the training set accuracy of classification was 95%. Since the data used in the work is unbalanced the accuracy of prediction may not be the best measure [1,28].

In order to address this problem a method proposed [51] to estimate the geometric mean G_M _(equation (15)) is used, whereas, *S*_*T *_(sensitivity) and *S*_*P *_(specificity) are estimated using equations (16) and (17), respectively.

(15) $$G_{M}=\sqrt{S_{T}*S_{P}}$$

(16) $$S_{T}=\frac{t_{p}}{\left(t_{p}+f_{n}\right)}$$

Where *t*_*p *_and *f*_*n *_stands for *true positives *and *false negatives*, respectively.

(17) $$S_{P}=\frac{t_{n}}{\left(t_{n}+f_{p}\right)}$$

Where *t*_*n *_and *f*_*p *_stands for *true negatives *and *false positives*, respectively.

**Figure 7:**
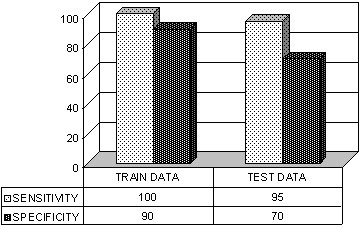
**Sensitivity and specificity**. Sensitivity and specificity obtained from the training set classification results and testing set classification results

**Figure 8:**
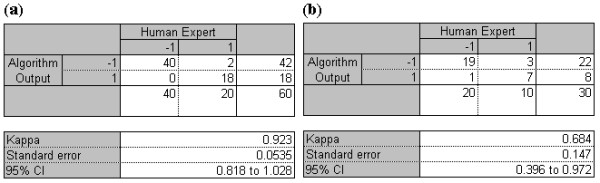
**Estimation of Kappa values**. Presents confusion matrix and kappa value for the (a) training set, (b) testing set

The *S*_*T *_percentage obtained for the training data classification was 100% and *S*_*P *_was 90%, and for the testing data set classification *S*_*T *_was 95% and *S*_*P *_was 70%, as shown in Figure 7. The *t*_*p*_, *t*_*n*_, *f*_*p*_, and *f*_*n *_values used for estimating *S*_*T *_and *S*_*P *_of the training and the testing data sets are taken from the confusion matrix shown in Figure 8a and Figure 8b, respectively. From *S*_*T *_and *S*_*P *_values the geometric mean G_M _was estimated according to equation (15) [1,28], for both training and testing data: 94.87 (training set) and 81.55 (testing set).

In order to eliminate any agreement arrived by chance, the results of the SVM classifier and the mutually agreed visual interpretation results from the two experienced obstetricians were compared using inter-rater agreement Kappa statistics [50,52,6]. The kappa value *k *is estimated using equation (18).

(18) $$k=\frac{p(a)-p(e)}{1-p(e)}$$

Here, *p(a) *is the relative observed agreement and *p(e) *is the hypothetical probability of agreement by chance. A 95% confidence interval (CI) is used in this work as shown in Figure 8. In Figure 8a and 8b the kappa value 0.923 with 95% CI was obtained for the training set and 0.684 for the testing set, respectively. The kappa values presented show a good agreement between the mutually agreed visual interpretation results from the two experienced obstetricians and the SVM classifier results, for both testing and training data.

## Discussion

The proposed methodology showed that the statistical features extracted from the decomposed components using EMD yields good classification performance (based on kappa values). It is difficult to make direct comparison of the proposed method with others as the approaches are different. However, based on the methods employed for statistical/mathematical feature extraction and classification an indirect comparison of the proposed method with those developed by other researchers is provided. Thuner *et al *[53] employed wavelet transform based on the standard deviation corresponding to information contained in the coefficients *4*^*th *^and *5*^*th*^, and managed to achieve a complete separation between the class of healthy adults and adults with cardiac pathology.

Salamalekis *et al *[26] in their intrapartum study of diagnosing fetal hypoxia, from 10-minutes FHR patterns, used only the *2*^*nd*^*, 3*^*rd *^and *4*^*th *^wavelet coefficients and obtained a sensitivity of 97.9% and specificity of 83.3%. The use of fetal pulse oximetry may have contributed for the high performance value of their methodology. Georgoulas *et al *[48] considered standard deviations corresponding to all the six wavelet coefficients as input features and achieved an overall classification performance of 90% for 3 minutes window. They also [1] used features based on the entropy measure of the wavelet coefficients and obtained a maximum geometric mean of 83.67% with a sensitivity of 75% and a specificity of 93.33% on 5-minutes segment. In all of these works [1,48,26] FHR traces were associated to umbilical artery pH values and since there is no consensus (gold standard) regarding its threshold, different values were considered to discriminate normal fetuses from those at risk. All these studies very concerned with the prediction of metabolic acidosis during the intrapartum period. More recently, Kampouraki *et al *[49] extracted features using statistical methods and signal analysis methods (Wavelet Transform) but from the adult heart rate and achieved an accuracy of 100%.

In our study employing statistical features extracted based on EMD, no single feature could be identified that was capable of discriminating the 'normal' from 'at risk' classes, therefore all the statistical features were considered as inputs to the SVM classifier. With 20 minutes FHR signals, a sensitivity of 100% and specificity of 90% were achieved for the training set. Whereas, the testing set classification rates showed significant difference in the value of sensitivity (95%) and specificity (70%), even though the penalty parameters were set to handle the burden on the high false positive rate because of the imbalance nature of the data set used. SVM classifier has been successfully used in FHR feature classification [1,28,48,30,29,31], but most of the work in this field is based on extracting morphological features and providing the classification using SVM.

## Conclusion

A new method of statistical feature extraction from FHR signals using EMD is proposed in this work. The features extracted from the decomposed components were further classified as 'normal' and 'at risk' by the SVM classifier.

Because of the lack of gold standards in evaluating the performance of intelligent systems [54] the proposed method was validated using the mutually agreed visual classification results from two experienced obstetricians. Validation of intelligent approaches based on the visual interpretation of a team of obstetricians has been used in evaluating several systems [6,54,23]. A high inter-observer and intra-observer variability in visual inspection is reported in the literature; however, it is important to note that it is largely dependent on the expertise and experience of the clinician involved [6]. Therefore, visual classification results from obstetricians with over 20 years experience were used in this study.

The inter-rater agreement kappa values obtained for the training set (0.923) and the testing set (0.684) showed good agreement of the proposed methodology with the mutually agreed visual classification results of two experts. This proved the viability of the method and its potential for further application.

The major limitations of this method are: the sifting process used in EMD is time consuming and the number of decomposed components varies with respect to the signal resulting in some empty spaces in the feature set. The preprocessing stage was helpful in removing the noisy components (IMFs). At this stage we can state that the results are quite promising. However, to be of clinical significance the proposed methodology requires extensive validation on a bigger data set.

As a future work, the proposed methodology would be applied to FHR signals of different durations and also would be extended for multi-class classification (here only two class labels were considered). Another possible extension is the classification of FHR by associating the traces to apgar scores. The effect of different sampling rates on features extracted using EMD and its effect on the SVM classifier also need further investigation.

## Competing interests

The authors declare that they have no competing interests.

## Authors' contributions

NKB was involved in the conception, design, development and analysis of the system and also drafted the manuscript. MAMA participated in the conception, design and analysis of the system, and helped in drafting the manuscript. ZE was involved in the conception of the study and provided critical review that helped in improving the manuscript. SA participated in conception, data acquisition and interpretation of FHR data. HFM was involved in the FHR data interpretation and analysis. All authors read and approved the final manuscript.
